# M. Oesterlen on the Passage of Metallic Mercury into the Blood and Various Organs of the Body

**Published:** 1845-01

**Authors:** 


*Passage of Metallic Mercury into the Blood and various Organs of the Body.*—M. Oesterlen has performed a number of experiments on animals, with the view of determining this point. The results of these are as follow:—

1. It is indubitable that mercury may pass, in the metallic state, through the parietes of the blood-vessels, since minute globules of it have been found in the subcutaneous cellular tissue and in the veins permeating it. The globules have never been discovered in the epidermic layers, cut only in the deep-seated layers of the dermis, near the blind extremities of the hair-follicles; also in these follicles and in the sudoriferous canals. 2. The metallic mercury, rubbed on the skin or introduced into the intestinal canal, may give rise to injurious effects, by passing into the current of the circulation. It is not easy to determine in what manner the metallic mercury, when once introduced into the circulation, becomes changed and modified, or how it then acts. At the side of the shining globules, M. Oesterlen always found a number of dull and dark-coloured corpuscles, which resembled a good deal the granules of a mercurial oxyde; these were found to be not acted upon by alkalis, but to be dissolved slowly in strong nitric acid, after being ground down into a fine powder. In the urine and in the bile, the mercurial globules did not exhibit any appearance of decided change. 3. Minute globules of this metal, in the state of fine division, may traverse the capillaries without producing any inflammatory *stasis:* their presence in the vessels does not seem to influence the formation of the blood or the development of the sanguineous corpuscles. 4. Small quantities of mercury, taken inwardly or applied on the skin, appear to pass chiefly into the parenchymatous substance of the spleen, liver and kidneys, and to be discharged by the last two emunctories.

M. Oesterlen has never been able to detect the presence of any globules in the *cells* of the liver, or in the corpuscles of the spleen, or in the minute tubercles of the kidney: they were always on the outer surface of these organs. He conjectures that they may have escaped from the anatomical preparation, after it had been put up. He has, however, detected them in the saliva and also in the urine.—*Med. Chir. Rev. from Roser's Archives.*

